# Risk-Based Colposcopy for Cervical Precancer Detection: A Cross-Sectional Multicenter Study in China

**DOI:** 10.3390/diagnostics12112585

**Published:** 2022-10-25

**Authors:** Peng Xue, Samuel Seery, Qing Li, Yu Jiang, Youlin Qiao

**Affiliations:** 1 Department of Epidemiology and Biostatistics, School of Population Medicine and Public Health, Chinese Academy of Medical Sciences and Peking Union Medical College, Beijing 100730, China; 2 Division of Health Research, Faculty of Health and Medicine, Lancaster University, Lancaster LA1 4YW, UK; 3 Diagnosis and Treatment for Cervical Lesions Center, Shenzhen Maternity & Child Healthcare Hospital, Shenzhen 518028, China

**Keywords:** cervical cancer screening, colposcopy, precancer, risk assessment

## Abstract

Recently published guidelines depend upon screening for cervical precancer risk stratification; however, colposcopy provides key information. There is no data from developing countries that could be used comparatively. Therefore, we assessed the potential benefits of intercalating colposcopic impressions with screening results to detect cervical precancers through a multicenter, cross-sectional study of a Chinese population. Anonymized data from 6012 women with cytologic assessment, human papillomavirus (HPV) testing, colposcopic impressions, and histological results were analyzed. Univariate and multivariate analysis showed that high-grade squamous intraepithelial lesion (HSIL) cytology, HPV16/18+, and/or high-grade colposcopic impressions markedly increased cervical precancer risk, while high-grade colposcopic impressions were associated with the highest risk. The risk of cervical intraepithelial neoplasia grade 3 or worse (CIN3+) ranged from 0% for normal/benign colposcopic impressions, <HSIL cytologies, and HPV negative to 63.61% for high-grade colposcopy, HSIL+ cytology, and HPV16/18+, across 18 subgroups. High-grade colposcopic impressions were associated with a >19% increased risk of CIN3+, even in participants without HSIL+ cytology and/or HPV16/18+. Regardless of screening outcomes, normal/benign colposcopic impressions were associated with the lowest risk of CIN3+ (<0.5%). Integrating colposcopic impressions into risk assessment may therefore provide key information for identifying cervical precancer cases. Adopting this approach may improve detection rates while also providing reassurance for women with a lower risk of developing cervical cancer.

## 1. Introduction

Cervical cancer is the fourth most prevalent cancer among women, and this presents a substantial problem for populations in low- and middle-income countries (LMICs). The World Health Organization has called on all nations to scale-up the implementation of proven, effective strategies to eradicate cervical cancer by 2030 [1]. These strategies include vaccinating 90% of all girls against human papillomavirus (HPV) by 15 years of age, screening 70% of all women, and identifying and treating 90% of all cases to manage precancers/cancers. Currently, potential cases are referred to a colposcopist for assessment to confirm the presence of precancers/cancers, which may then lead to biopsy or treatment [2,3,4]. Therefore, colposcopy is of vital importance in the diagnostic pathway and should be investigated for potential improvements.

The American Society of Colposcopy and Cervical Pathology (ASCCP) recently introduced quantitative risk estimates to adjust patient management techniques depending on precancer risk [5,6,7]. However, the guidelines depend solely upon screening results for cytology and/or HPV and are geared toward the detection of intraepithelial neoplasia grade 3 or worse (CIN3+). This is where colposcopy can provide key information for risk assessment and enhance the ability to identify cases. Several researchers have considered the feasibility of this approach using United-States–based datasets [8,9,10]. However, there is no comparative data available from LMICs, which face a greater burden related to cervical cancer. Therefore, we sought to assess the potential benefit of intercalating colposcopic impressions with screening test results (cytology and HPV testing) for cervical precancer detection in Chinese clinical settings using available registry data. 

## 2. Materials and Methods

### 2.1. Study Participants

This study was conducted between January 2019 and October 2021 in six different colposcopy clinics across China. We collated registry data from women who visited a colposcopist. All participants were Chinese nationals. Patients older than 18 years of age and those who had been referred to a local colposcopist because of abnormal screening results or those with symptoms of suspected cervical disease were eligible to participate. Women who underwent a hysterectomy or previous treatment for cervical lesions and those who had undergone biopsies for cervical lesions without a definitive pathological diagnosis were excluded. Anonymized digital patient records, which included cytology, HPV testing, colposcopic impressions, and histological results, were collected. 

This study was performed in accordance with the Declaration of Helsinki ethical standards and was approved by the Institutional Review Board (IRB) of the Chinese Academy of Medical Sciences and Peking Union Medical College (CAMS/PUMC). The need for informed consent was waived because this was a retrospective study of pre-existing clinical archives and because all data were completely anonymized.

### 2.2. Cytology and HPV Testing

Abnormal screening results from cytology or/and HPV testing were the main referral condition for colposcopy. In accordance with the Bethesda system [11], we categorized participants as either <HSIL or HSIL+: <HSIL included those negative for intraepithelial lesion or malignancies (NILM), atypical squamous cells of undetermined significance (ASC-US), and low-grade squamous intraepithelial lesion (LSIL); HSIL+ included those with atypical squamous cells that did not exclude high-grade squamous intraepithelial lesion (ASC-H), high-grade squamous intraepithelial lesion (HSIL), squamous cell carcinoma (SCC), adenocarcinoma in situ (AIS), or atypical glandular cells (AGC). The type of HPV test used was not recorded because it was not considered pertinent to this study. High-risk HPV types were defined as HPV16/18, non-16/18 hrHPV, or hrHPV-negative. 

### 2.3. Colposcopy and Biopsy

Colposcopic examinations were performed by experienced colposcopists in hospitals; all colposcopists were accredited by the Chinese Society of Cervical Pathology and Colposcopy. International standards stipulate that qualified colposcopists should be trained and certified by a professional colposcopy training institution, have at least one year of experience and manage at least 100 newly diagnosed cases per year [12]. Colposcopic impressions were graded and distinguished following the classification of the International Federation of Cervical Pathology and Colposcopy (IFCPC) [13] as suspicious cancer, high-grade, low-grade, or normal/benign. Suspicious cases were also referred to as “high-grade” colposcopic impressions. All colposcopically detected abnormalities were directly biopsied. If no visible lesions were observed, a random biopsy was performed at the squamocolumnar junction in four quadrants, positionally at 2, 4, 8, or 10 o’clock. If necessary, an endocervical curettage was performed after cervical biopsies.

### 2.4. Histological Diagnosis

Histological reports of biopsy and excision specimens were reviewed by a panel of experienced pathologists from the six hospitals involved in the study. Disagreements were resolved through consultation with independent pathologists. Histological diagnoses were categorized as cervical intraepithelial neoplasia (CIN) by order of severity: normal; CIN grade 1, 2, 3, and adenocarcinoma; normal CIN with a negative biopsy; negative hrHPV results; and cytology showing either NILM or ASC-US. CIN2+ included patients with a histological diagnosis of CIN grade 2, 3, or adenocarcinoma [14]. CIN3+ included patients with a histological diagnosis of CIN3 or adenocarcinoma. When analyzing biopsies, excision specimens, and/or endocervical curettage together, the final histological diagnosis was considered the worst grade of dysplasia present.

### 2.5. Statistical Analysis

Two cervical precancer endpoints (CIN2+ and CIN3+) were defined for risk assessment. CIN3+ was established as the main threshold. CIN2+ may be considered suboptimal for these purposes and generally has poor pathological reproducibility and greater regression even without any treatment [15,16]. We considered all possible risk stratifications based on combinations of colposcopic impressions and screening results (cytology and/or HPV status). There were six subgroups of colposcopic impressions with cytology, nine subgroups for colposcopic impressions with HPV status, and 18 subgroups of colposcopy impressions with cytology and HPV status. For each subgroup, the immediate risk of histological CIN2+ or CIN3+ was calculated by dividing the number of CIN2+ or CIN3+ cases in all women in each category. Risk was reported as a frequency percentage. Univariate and multivariate logistic regression models were developed to assess independent factors that were reported as odds ratios (OR), with 95% confidence intervals (CI) for CIN2+ and CIN3+. All analyses were conducted using SAS software, version 9.4. A *p* value < 0.05 was set as the threshold for statistical significance.

### 2.6. List of Risk-Based Strata Assessed

We assessed the following risk strata in all possible combinations:

1. Colposcopy and cytology (six subgroups)
  1a. High-grade colp and HSIL+
  1b. High-grade colp and <HSIL
  1c. Low-grade colp and HSIL+
  1d. Low-grade colp and <HSIL
  1e. Normal/benign colp and HSIL+
  1f. Normal/benign colp and <HSIL
2. Colposcopy and HPV status (nine subgroups)
  2a. High-grade colp and HPV16/18
  2b. High-grade colp and non-16/18 hrHPV
  2c. High-grade colp and hrHPV negative
  2d. Low-grade colp and HPV16/18
  2e. Low-grade colp and non-16/18 hrHPV
  2f. Low-grade colp and hrHPV negative
  2g. Normal/benign colp and HPV16/18
  2h. Normal/benign colp and non-16/18 hrHPV
  2i. Normal/benign colp and hrHPV negative
3. Colposcopy, cytology and HPV status (18 subgroups)
  3a. High-grade colp and HSIL+ and HPV16/18+
  3b. High-grade colp and HSIL+ and non-16/18 hrHPV
  3c. High-grade colp and HSIL+ and hrHPV negative
  3d. High-grade colp and <HSIL and HPV16/18+
  3e. High-grade colp and <HSIL and non-16/18 hrHPV
  3f. High-grade colp and <HSIL and hrHPV negative
  3g. Low-grade colp and HSIL+ and HPV16/18+
  3h. Low-grade colp and HSIL+ and non-16/18 hrHPV
  3i. Low-grade colp and HSIL+ and hrHPV negative
  3j. Low-grade colp and <HSIL and HPV16/18+
  3k. Low-grade colp and <HSIL and non-16/18 hrHPV
  3l. Low-grade colp and <HSIL and hrHPV negative
  3m. Normal/benign colp and HSIL+ and HPV16/18+
  3n. Normal/benign colp and HSIL+ and non-16/18 hrHPV
  3o. Normal/benign colp and HSIL+ and hrHPV negative
  3p. Normal/benign colp and <HSIL and HPV16/18+
  3q. Normal/benign colp and <HSIL and non-16/18 hrHPV
  3r. Normal/benign colp and <HSIL and hrHPV negative

## 3. Results

A total of 6012 women with abnormal screening results or clinical symptoms of suspected cervical diseases who were referred to colposcopy were included in this study. The demographics and clinical information of all participants are provided in Table 1. Approximately 65.42% (*n* = 3933) of participants were 30–49 years of age, which is within the recommended age range for cervical cancer screening. In the total group, 12.31% (*n* = 740) had HSIL+ cytology results, 34.08% (*n* = 2049) had HPV16/18+ infection, and 21.37% (*n* = 1285) had high-grade colposcopic impressions. Histological analysis revealed that 75.57% (*n* = 4543) of samples were <CIN2, that 14.65% (*n* = 881) were CIN2, and that 8.65% (*n* = 520) were CIN3; cancer was found in 1.13% (*n* = 68) of samples.

### 3.1. Associations between Colposcopy, Cytology HPV Status, and Precancer Diagnosis

The associations between colposcopy, cytology HPV status, and the histology diagnosis of CIN2+ and CIN3+ using univariate and multivariate logistical regression analysis are shown in Table 2. In both univariate and multivariate analysis, HSIL+ cytology, HPV 16/18+, and high-grade colposcopic impressions were significantly related to an increased risk of CIN2+. However, the risk associated with high-grade colposcopic impressions (OR: 133.71, 95% CI: 95.63–186.95, *p* < 0.001; OR: 70.03, 95% CI: 49.43–99.19, *p* < 0.001) was substantially higher than HSIL+ cytology (OR: 11.39, 95% CI: 9.57–13.55, *p* < 0.001) and OR: 3.54, 95% CI: 2.79–4.51, *p* < 0.001). HPV 16/18+ also had an OR: 10.54, 95% CI: 7.95–13.97 (*p* < 0.001) and OR: 4.91, 95% CI: 3.41–7.07 (*p* < 0.001). Similar results were observed in individuals at risk of CIN3+.

### 3.2. Cervical Precancer Risk Strata Based on Colposcopy and Cytology

When HPV genotyping was not available in clinical scenarios, we considered risk estimates using only colposcopic impressions (i.e., high-grade, low-grade, and normal/benign) and referral cytology (HSIL+ and <HSIL). Data from each participant were categorized into one of six subgroups. Table 3 and Figure S1 show the categories with the numbers of women and the number of both CIN2+ and CIN3+ outcomes ranked by risk. The risk of histological CIN2+ ranged from 0% in the lowest risk subgroup (normal/benign colposcopy, HSIL+ cytology) to 89.02% for the highest risk subgroups (high-grade colposcopy, HSIL+ cytology).

The risk of histological CIN3+ ranged from 0% for normal/benign colposcopy, HSIL+ cytology in the lowest subgroup to 53.76% for the highest risk subgroups (high-grade colposcopy, HSIL+ cytology). Women with high-grade colposcopic impressions were at the highest risk of CIN2+ (more than 72%), and the risk of CIN3+ was more than 30%, even in those with cytologic results that did not suggest the presence of HSIL+. Irrespective of cytological outcomes, women with normal/benign colposcopic impressions had a risk of <3% for CIN2+ and <1% for CIN3+.

### 3.3. Cervical Precancer Risk Strata Based on Colposcopy and HPV Status

When cytology was not available in clinical scenarios, we considered colposcopic impressions (high-grade, low-grade and normal/benign) and referral HPV genotyping testing (hrHPV negative, non-16/18 hrHPV and HPV16/18). Participants were then assigned to one of nine subgroups. Table 4 and Figure S2 show the categories with the numbers of women and the number of both CIN2+ and CIN3+ outcomes ranked by risk. The risk of histological CIN2+ ranged from 0.90% in the lowest-risk subgroup (normal/benign colposcopy, hrHPV negative) to up to 85.33% in the highest-risk subgroup (high-grade colposcopy, HPV16/18+). The risk of histological CIN3+ ranged from 0% (normal/benign colposcopy, hrHPV negative) in the lowest-risk subgroup to up to 48.23% in the highest-risk subgroup (high-grade colposcopy, HPV16/18+). Women with high-grade colposcopic impressions showed a higher risk of CIN2+, greater than 60%, and a risk for CIN3+ greater than 27%. This finding was consistent even if HPV genotyping did not suggest the presence of HPV16/18. Irrespective of HPV genotyping, women with normal/benign colposcopies were at low risk for CIN2+ (<6%) and CIN3+ (<1%).

### 3.4. Cervical Precancer Risk Strata Based on Colposcopy, Cytology, and HPV Status

**Figure 1:**
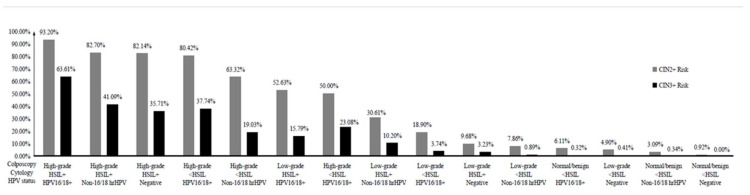
The sequence of cervical precancer risk strata based on colposcopy cytology and HPV status. Abbreviations: CIN2+, cervical intraepithelial neoplasia grade 2 or worse; CIN3+, cervical intraepithelial neoplasia grade 3 or worse; <HSIL, includes negative for intraepithelial lesion or malignancy (NILM), atypical squamous cells of undetermined significance (ASC-US), low-grade squamous intraepithelial lesion (LSIL); HSIL+, high-grade squamous intraepithelial lesion or worse; HPV, human papillomavirus; hrHPV, high-risk HPV.

We assigned each participant to one of 18 subgroups on the basis of the findings of the three tests. Table 5 and Figure 1 show details on the categories with the number of women and the number of both CIN2+ and CIN3+ outcomes ranked by risk. The proportion of women in each subgroup varied considerably, ranging from 93.20% for those with high-grade colposcopic impressions, HSIL+ cytology, and HPV16/18+ to 0% for those with a normal/benign colposcopy, HSIL+ cytology, and hrHPV negative. Seven subgroups had pooled CIN2+ risk estimates above 50%: high-grade colposcopy impression, HSIL+ cytology, and HPV16/18+; high-grade colposcopy impression, HSIL+ cytology, and non-16/18 hrHPV; high-grade colposcopy impression, HSIL+ cytology, and hr-HPV negative; high-grade colposcopy impression, <HSIL cytology, and HPV16/18+; high-grade colposcopy impression, <HSIL cytology, and non-16/18 hrHPV; low-grade colposcopy impression, HSIL+ cytology, and HPV16/18+; and high-grade colposcopy impression, <HSIL cytology, and hr-HPV negative.

Pooled risk estimates for CIN3+ had similar patterns to CIN2+. There was also a correlation between high-risk subgroups and histological CIN2+ and CIN3+ risk: women with high-grade colposcopic impressions were more likely to have HSIL cytology and/or HPV16/18+. Women with high-grade colposcopic impressions showed a high risk for CIN2+ (50%) and CIN3+ (≥19%), even in those with screening results that did not suggest the presence of HPV16/18+ or HSIL+ cytology. Again, irrespective of the screening results, women with normal/benign colposcopies had a low risk for CIN2+ (<7%) and CIN3+ (<1%).

## 4. Discussion

Based on standard colposcopic practice in six centers across China, we found that HSIL cytology, HPV16/18+, and high-grade colposcopic impressions appear to be significantly related to both CIN2+ and CIN3+. These findings were consistent under both univariate and multivariate analyses and were increasingly evident when colposcopic impressions highly correlated. This suggests that colposcopy may be useful for CIN2+ and CIN3+ risk stratification in China. This is an important finding that advances our knowledge for clinical practice and education. In a previous systematic review, researchers studied the effectiveness of a pooled risk-based tool that combines three testing results (cytology, HPV16/18, and colposcopic impressions) for cervical precancer detection [17]. However, the data was limited because few researchers in the original studies examined the pooled risk of precancers. Therefore, few of the included studies intercalated all three tests, which influenced separate sample sizes. Additionally, many of the included studies chose a suboptimal CIN2+ endpoint as the threshold, which has poor pathological reproducibility and usually has a greater regression to the mean, even in those who did not receive treatment. 

Herein, we conducted primary research and incorporated different thresholds that enabled us to analyze data from different perspectives. All possible risk stratifications were assessed using combinations of colposcopic impressions and cytology or/and HPV testing. We found that women with high-grade colposcopic impressions had a CIN2+ risk ranging from 50.0% to 93.20%. We also found that CIN3+ risk ranged from 19.03% to 63.19%, even among women who had an initial low-risk screening result, i.e., <HSIL cytology and/or non-16/18 hrHPV infection or hr-HPV negative. By contrast, normal/benign colposcopic impressions showed risks of CIN2+ ranging from 0% to 6.11% and CIN3+ risks ranging from 0% to less than 0.5%, even among women who were considered at high-risk, i.e., HPV16/18+. Our results suggest that there are similarities in low-risk patterns with normal/benign colposcopic impressions despite having different referral cytology and/or HPV testing results. Therefore, the low prevalence of CIN3+ in low-risk patients suggests that follow-up monitoring should be conducted in these women instead of biopsies, as previous reports have suggested [17,18]. 

Additionally, some combinations were not rare and were only occasionally detected, such as women with normal/benign colposcopic impressions, HSIL+ cytology, and HPV16/18+. This finding was again consistent with previous reports from clinical studies [19,20,21]. It is worth noting that our data come from experienced colposcopists in clinical settings in China. These findings therefore may not apply to other LMIC populations because training and skills may not be comparable. In China, our evidence appears to support the notion that cervical precancer risk assessment should intercalate colposcopic impressions. Additionally, women with at least two high-risk results from high-grade colposcopic impressions, HSIL+ cytology, and HPV16/18+, should be considered at highest risk because of the high prevalence of CIN2+ and CIN3+. Further research into 5- and 10-year recurrence rates following CIN2/3 treatment would be useful for follow-up scheduling using risk-based colposcopy. This may also provide more insight into the effectiveness of follow-up scheduling and could aid in the development of an artificial intelligence model that incorporates an initial screening step such as liquid-based cytology and HPV testing with lifestyle questionnaires before risk-based colposcopy for precancers [22].

The new ASCCP guidelines [5,6,7] suggest that there may be a need to expedite treatment without colposcopy-based biopsy. This is for women with HSIL and HPV16/18+ because the risk of CIN3+ is higher than 60%. The results from this study did not contradict these findings. We found that among Chinese women with high-grade colposcopic impressions (HSIL+ cytology and HPV16/18+) the prevalence of CIN3+ was 63.61%. In women with low-grade colposcopic impressions (HSIL+ cytology and HPV16/18+), the risk was considerably lower at 15.79%. This raises a number of questions around the immediacy of treatment that the ASCCP has tried to address. However, colposcopic assessment with biopsy should be the preferred strategy to ensure against the over-treatment of patients or causing unnecessary stress. The screening process impacts the emotions, self-esteem, and overall well-being of individuals. Various studies in the US, Germany, and Japan have highlighted the need for more psychosocial support throughout the cervical cancer screening process [23,24], and it is reasonable to assume that Chinese women encounter similar psychosocial issues. Therefore, reducing over-treatment can only be positive.

This study also found that, for women with HSIL+ cytology and HPV16/18+, the optimal treatment strategy may be different for women with high-grade colposcopic impressions compared with those thought to be at low-grade risk. This somewhat contradicts the “one-size-fits-all” approach to expediting treatments, as recommended in the most recent guidelines. The decision to intervene using invasive strategies should be modified by underlying risk. Women with normal/benign colposcopic impressions, <HSIL+ cytology, and non-hrHPV 16/18 negative or hr-HPV negative, were at lower risk of CIN2+, ranging from 0.92% to 3.09%, and CIN3+, ranging from 0% to 0.34%. This finding supports previous studies [9,10,17,18], which suggested that colposcopy-based biopsies are not recommended for underlying precancer identification. Therefore, adding colposcopy into standardized precancer risk assessment is likely to improve clinical practice. However, experienced colposcopists are not always readily available in LMICs. This also means that colposcopy training is not always standardized, and experience dramatically influences a physician’s performance.

In this study, we applied a risk assessment strategy recommended by National Cancer Institute (NCI) protocol [17]. However, risks vary on the basis of geographic regions [25]. Notably, we cannot determine risk based solely upon location because there are subtle differences between urban and rural risk. There are also a number of sub-cultures that may or may not be reported, adding risk and lifestyle elements that are not always considered. In this study, we attempted to gather data from a variety of sources that may provide more insight into the complexities of Chinese society. We included data from women visiting colposcopists in six different regions of mainland China. We hope this evidence will contribute to the development of risk-based management algorithms for Asian populations and may help initiate research into the subtle differences between and within global populations. Cervical cancer has heterogeneous variations in terms of metastatic spread, gene expression, and responses to treatments. Therefore, any well-designed research into cervical precancer screening and detection has the potential to provide valuable insights for population-specific disease prevention and health protection.

To the best of our knowledge, this is the first study to assess the potential benefit of intercalating colposcopic impressions with screening test results (cytology and HPV testing) for cervical precancer detection in Chinese population. However, the study has some limitations that must be considered. First, our findings only reflect the immediate risk of precancers. This study did not examine the course of development, which demands more attention. A longer follow-up period and screening histories and diagnosis will provide important information that will likely increase the precision of risk assessments. Second, we did not stratify patients by age. Several studies have suggested that refining risk-based assessment tools may also require factoring in age. We are conducting a related risk-stratification study to understand the effect of age on disease detection, and we hope to shed light on some of these issues. 

## 5. Conclusions

This study suggests that colposcopy practice could play an important role in assessing patients for their risk of underlying cervical precancers. We found that adding colposcopic impressions to risk-stratification procedures improves case identification. This approach improves the detection of cervical precancers in individuals at highest risk and may also reduce over-treatment, which can have a negative impact on women’s health and well-being. This intercalated approach may also provide reassurance for patients receiving normal/benign colposcopy results who are at lower risk. Our findings support the recommendation against non-directed biopsies for women with normal/benign colposcopic impressions, <HSIL cytology, and hrHPV negative status and suggests that there is a need for directed biopsies for women with high-grade colposcopic impressions, regardless of screening outcomes.

## Figures and Tables

**Table 1:** Baseline demographic and clinical information.

Characteristic	Number (%)
Age groups, y	
<30	989 (16.45)
30–39	2261 (37.61)
40–49	1672 (27.81)
>50	1090 (18.13)
Menopause	
No	5065 (84.25)
Yes	947 (15.75)
Gravidity	
None	584 (9.71)
1	1022 (17.00)
2	1685 (28.03)
3	1365 (22.70)
4+	1356 (22.56)
Parity	
None	944 (15.70)
1	2588(43.05)
2	1935 (32.19)
3+	545 (9.06)
Cytology	
<HSIL	5159 (85.81)
HSIL+	740 (12.31)
Not performed	113 (1.88)
HPV status	
hrHPV negative	889 (14.79)
Non-16/18 hrHPV	3035 (50.48)
HPV16/18+	2049 (34.08)
Not performed	39 (0.65)
Colposcopy impression	
Normal/benign	1501 (24.97)
Low-grade	3226 (53.66)
High-grade	1285 (21.37)
Histological outcome	
<CIN2	4543 (75.57)
CIN2	881 (14.65)
CIN3	520 (8.65)
Cancer	68 (1.13)

Abbreviations: <HSIL, includes negative for intraepithelial lesion or malignancy (NILM), atypical squamous cells of undetermined significance (ASC-US), low-grade squamous intraepithelial lesion (LSIL); HSIL+, high-grade squamous intraepithelial lesion or worse; HPV, human papillomavirus; hrHPV, high-risk human papillomavirus; <CIN2, includes normal, cervical intraepithelial neoplasia grade 1; CIN2, cervical intraepithelial neoplasia grade 2; CIN3, cervical intraepithelial neoplasia grade 3.

**Table 2:** Association between colposcopy, cytology and HPV status and the diagnosis of cervical precancers.

Risk Factors	CIN2+				CIN3+			
	Univariate Analysis		Multivariate Analysis		Univariate Analysis		Multivariate Analysis	
	OR (95% CI)	*p*	OR (95% CI)	*p*	OR (95% CI)	*p*	OR (95% CI)	*p*
Cytology								
<HSIL	1 (Reference)		1 (Reference)		1 (Reference)		1 (Reference)	
HSIL+	11.39 (9.57–13.55)	<0.001	3.54 (2.79–4.51)	<0.001	11.81 (9.77–14.29)	<0.001	2.87 (2.27–3.62)	<0.001
HPV status								
hrHPV negative	1 (Reference)		1 (Reference)		1 (Reference)		1 (Reference)	
Non-16/18 hrHPV	3.12 (2.35–4.15)	<0.001	1.86 (1.29–2.67)	0.001	2.68 (1.63–4.38)	<0.001	1.27 (0.72–2.22)	0.41
HPV16/18+	10.54 (7.95–13.97)	<0.001	4.91 (3.41–7.07)	<0.001	11.74 (7.27–18.96)	<0.001	3.65 (2.11–6.33)	<0.001
Colposcopy								
Normal/benign	1 (Reference)		1 (Reference)		1 (Reference)		1 (Reference)	
Low-grade	5.02 (3.63–6.93)	<0.001	4.00 (2.87–5.58)	<0.001	11.72 (3.69–37.24)	<0.001	10.14 (3.18–32.40)	<0.001
High-grade	133.71 (95.63–186.95)	<0.001	70.03 (49.43–99.19)	<0.001	329.66 (105.62–1028.94)	<0.001	162.15 (51.37–511.79)	<0.001

Abbreviations: CIN2+, cervical intraepithelial neoplasia grade 2 or worse; CIN3+, cervical intraepithelial neoplasia grade 3 or worse; OR, odds ratio; CI, confidence interval; <HSIL, includes negative for intraepithelial lesion or malignancy (NILM), atypical squamous cells of undetermined significance (ASC-US), low-grade squamous intraepithelial lesion (LSIL); HSIL+, high-grade squamous intraepithelial lesion or worse; HPV, human papillomavirus; hrHPV, high-risk human papillomavirus.

**Table 3:** Cervical precancer risk strata based on colposcopy and cytology.

ID	Colposcopy	Cytology	Total, N	CIN2+, N	Risk	CIN3+, N	Risk
1a	High-grade	HSIL+	519	462	0.8902	279	0.5376
1b	High-grade	<HSIL	742	540	0.7278	224	0.3019
1c	Low-grade	HSIL+	187	63	0.3369	20	0.1070
1d	Low-grade	<HSIL	2975	329	0.1106	53	0.0178
1f	Normal/benign	<HSIL	1442	42	0.0291	3	0.0021
1e	Normal/benign	HSIL+	34	0	0	0	0

Abbreviations: CIN2+, cervical intraepithelial neoplasia grade 2 or worse; CIN3+, cervical intraepithelial neoplasia grade 3 or worse; <HSIL, includes negative for intraepithelial lesion or malignancy (NILM), atypical squamous cells of undetermined significance (ASC-US), low-grade squamous intraepithelial lesion (LSIL); HSIL+, high-grade squamous intraepithelial lesion or worse. Risk is reported as a frequency percentage. The risk of histological CIN2+ or CIN3+ was calculated by dividing the number of CIN2+ or CIN3+ threshold over all women in each stratum.

**Table 4:** Cervical precancers risk in strata based on colposcopy and HPV status.

ID	Colposcopy	HPV Status	Total, N	CIN2+, N	Risk	CIN3+, N	Risk
2a	High-grade	HPV16/18	736	628	0.8533	355	0.4823
2b	High-grade	Non-16/18 hrHPV	480	341	0.7104	131	0.2729
2c	High-grade	hrHPV negative	54	36	0.6667	16	0.2963
2d	Low-grade	HPV16/18	993	212	0.2135	44	0.0443
2e	Low-grade	Non-16/18 hrHPV	1942	176	0.0906	26	0.0134
2g	Normal/benign	HPV16/18	320	19	0.0594	1	0.0031
2f	Low-grade	hrHPV negative	277	16	0.0578	2	0.0072
2h	Normal/benign	Non-16/18 hrHPV	613	18	0.0294	2	0.0033
2i	Normal/benign	hrHPV negative	558	5	0.0090	0	0

Abbreviations: CIN2+, cervical intraepithelial neoplasia grade2 or worse; CIN3+, cervical intraepithelial neoplasia grade3 or worse; HPV, human papillomavirus; hrHPV, high-risk human papillomavirus. Risk is reported as a frequency percentage. The risk of histological CIN2+ or CIN3+ was calculated by dividing the number of CIN2+ or CIN3+ threshold over all women in each stratum.

**Table 5:** Cervical precancers risk strata based on colposcopy, cytology, and HPV status.

ID	Colposcopy	Cytology	HPV Status	Total, N	CIN2+, N	Risk	CIN3+, N	Risk
3a	High-grade	HSIL+	HPV16/18+	294	274	0.9320	187	0.6361
3b	High-grade	HSIL+	Non-16/18 hrHPV	185	153	0.8270	76	0.4109
3c	High-grade	HSIL+	hrHPV negative	28	23	0.8214	10	0.3571
3d	High-grade	<HSIL	HPV16/18+	424	341	0.8042	160	0.3774
3e	High-grade	<HSIL	Non-16/18 hrHPV	289	183	0.6332	55	0.1903
3g	Low-grade	HSIL+	HPV16/18+	57	30	0.5263	9	0.1579
3f	High-grade	<HSIL	hrHPV negative	26	13	0.50	6	0.2308
3h	Low-grade	HSIL+	Non-16/18 hrHPV	98	30	0.3061	10	0.1020
3j	Low-grade	<HSIL	HPV16/18+	910	172	0.1890	34	0.0374
3i	Low-grade	HSIL+	hrHPV negative	31	3	0.0968	1	0.0323
3k	Low-grade	<HSIL	Non-16/18 hrHPV	1807	142	0.0786	16	0.0089
3p	Normal/benign	<HSIL	HPV16/18+	311	19	0.0611	1	0.0032
3l	Low-grade	<HSIL	hrHPV negative	245	12	0.0490	1	0.0041
3q	Normal/benign	<HSIL	Non-16/18 hrHPV	582	18	0.0309	2	0.0034
3r	Normal/benign	<HSIL	hrHPV negative	541	5	0.0092	0	0
3n	Normal/benign	HSIL+	Non-16/18 hrHPV	17	0	0	0	0
3o	Normal/benign	HSIL+	hrHPV negative	16	0	0	0	0
3m	Normal/benign	HSIL+	HPV16/18+	None				

Abbreviations: CIN2+, cervical intraepithelial neoplasia grade2 or worse; CIN3+, cervical intraepithelial neoplasia grade3 or worse; <HSIL, includes negative for intraepithelial lesion or malignancy (NILM), atypical squamous cells of undetermined significance (ASC-US), low-grade squamous intraepithelial lesion (LSIL); HSIL+, high-grade squamous intraepithelial lesion or worse; HPV, human papillomavirus; hrHPV, high-risk human papillomavirus. Risk is reported as a frequency percentage. The risk of histological CIN2+ or CIN3+ was calculated by dividing the number of CIN2+ or CIN3+ threshold over all women in each stratum.

## Data Availability

The datasets generated and/or analyzed during the current study are not publicly available because of personal information protection, patient privacy regulation, and medical institutional data regulatory policies but are available from the corresponding author on reasonable request and with permission.

## Supplementary Materials

The following supporting information can be downloaded at: https://www.mdpi.com/article/10.3390/diagnostics12112585/s1, Figure S1: The sequence of cervical precancers risk strata based on colposcopy and cytology, Figure S2: The sequence of cervical precancers risk strata based on colposcopy and HPV status.
