# Supplementary material for: Risk-Based Colposcopy for Cervical Precancer Detection: A Cross-Sectional Multicenter Study in China
Source: Diagnostics (Basel). 2022 Oct 25;12(11):2585. doi: 10.3390/diagnostics12112585 (PMC9689887; doi:10.3390/diagnostics12112585)
Supplement: Supplementary file 1 [file diagnostics-12-02585-s001.zip › diagnostics-1976138-supplementary.pdf]

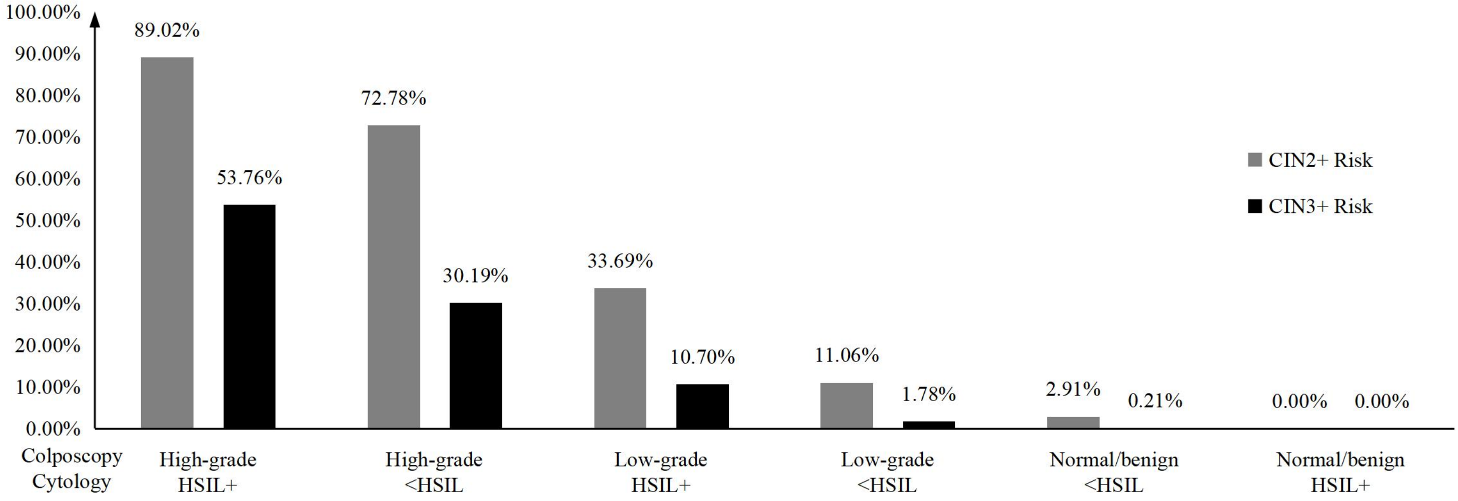

FIGURE S1 | The sequence of cervical precancers risk strata based on colposcopy and cytology. Abbreviations: CIN2+, cervical intraepithelial neoplasia grade2 or worse; CIN3+, cervical intraepithelial neoplasia grade3 or worse; <HSIL, includes negative for intraepithelial lesion or malignancy (NILM), atypical squamous cells of undetermined significance (ASC-US), low-grade squamous intraepithelial lesion (LSIL); HSIL+, high-grade squamous intraepithelial lesion or worse.

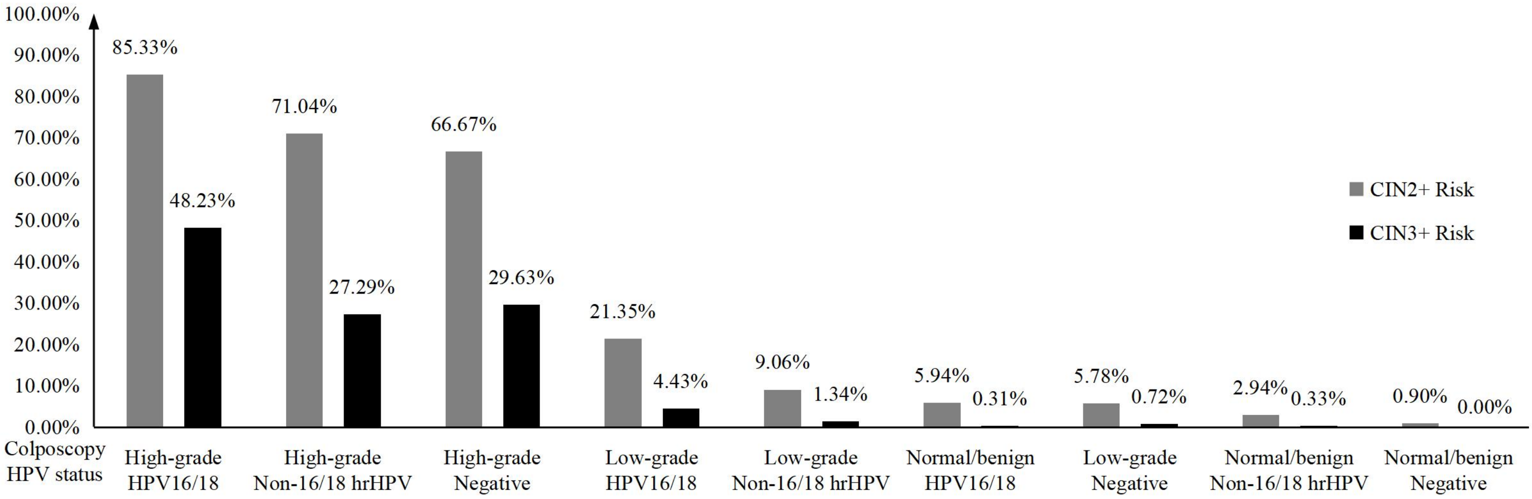

FIGURE S2 |The sequence of cervical precancers risk strata based on colposcopy and HPV status. Abbreviations: CIN2+, cervical intraepithelial neoplasia grade2 or worse; CIN3+, cervical intraepithelial neoplasia grade3 or worse; HPV, human papillomavirus; hrHPV, high-risk HPV.
